# Effect of Prenatal and Postnatal Stress in Rats on the Gut Microbiome in Adolescence

**DOI:** 10.1111/jnc.70530

**Published:** 2026-07-27

**Authors:** Rebecca Woods, Elliot F. Jennings, Laura Smith, Khairiah Almushri, Liam Hanson, Oriana Gamrot, Ayomide Adetunji, Chiamaka Vera Oguanya, Hamilton Imongan, Kubili John, Emmanuella Omuluche, Michael Harte, Chris Murgatroyd

**Affiliations:** ^1^ School of Biological and Chemical Sciences Manchester Met University Manchester UK; ^2^ Division of Evolution, Infection and Genomics, School of Biological Sciences, Faculty of Biology Medicine and Health University of Manchester Manchester UK; ^3^ Division of Pharmacy and Optometry, School of Health Sciences, Faculty of Biology Medicine and Health University of Manchester Manchester UK

**Keywords:** dexamethasone, microbiome, postnatal stress, prenatal stress, rat model

## Abstract

Previous research suggests that early‐life stress (ELS) increases the risk of mental health disorders later in life. It is hypothesised that ELS disrupts the developing gut microbiome, which in turn may alter neuroendocrine and immune system development, thereby increasing disease susceptibility. However, the specific microbial taxa and pathways mediating these effects remain poorly characterised. Here, we used rat models to investigate whether ELS leads to long‐term alterations in the gut microbiome. Microbial composition was assessed using 16S rRNA Nanopore sequencing of DNA extracted from faecal pellets of adolescent male and female rats exposed to: (i) early postnatal dexamethasone (DEXA; a synthetic glucocorticoid) or saline control, (ii) prenatal stress (PRS) and controls, or (iii) postnatal stress (POS) and controls. Microbiome structure was evaluated using richness, evenness, dominance and diversity indices. We show that ELS induces model‐specific and sex‐dependent changes in gut microbiome composition, primarily at the level of overall community structure rather than individual taxa. DEXA exposure produced the most consistent compositional signature, particularly in males, whereas PRS showed minimal detectable effects and POS exhibited a more heterogeneous response characterised by increased dispersion and limited taxonomic shifts. More broadly, these findings demonstrate that integrating beta‐diversity analyses with machine learning approaches can identify reproducible microbiome patterns associated with ELS, even in the absence of large taxonomic changes. Applying similar frameworks in larger and longitudinal cohorts will be important to determine how these subtle microbial signatures contribute to long‐term physiological outcomes.

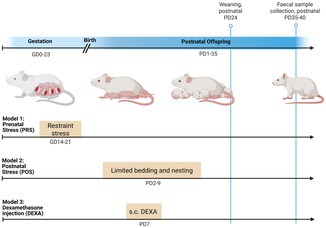

## Graphical abstract

Early‐life stress may raise the risk of mental health problems later in life. One possible reason is that stress affects the gut microbiome—the community of bacteria living in the digestive system—which can influence how the brain, hormones and immune system develop. In this study, we looked at rats exposed to different types of early stress and examined their gut bacteria. We found that stress changes the microbiome, shifting the overall balance and structure of the microbial community. These effects also varied depending on the type of stress and whether the rats were male or female. Interestingly, one type of stress (linked to a stress hormone drug) caused the most consistent changes, while others had weaker or more variable effects. Overall, the study suggests that early stress leaves subtle but detectable ‘fingerprints’ in the gut microbiome that might play an important role in long‐term health.

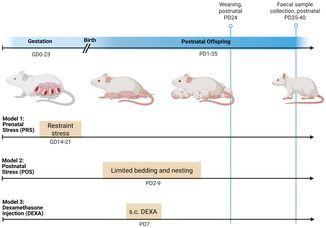

**Abbreviations**
CLR: centred log‐ratio
DEXA: dexamethasone
ELISA: enzyme‐linked immunosorbent assay
ELS: early‐life stress
FDR: false discovery rate
GD: gestational day
HPA: hypothalamic–pituitary–adrenal
ONT: Oxford Nanopore Technologies
PCoA: principal coordinate analysis
PD: postnatal day
PERMANOVA: permutational multivariate analysis of variance
PERMDISP: permutational analysis of multivariate dispersions
POS: postnatal stress
PRS: prenatal stress
RRID: Research Resource Identifier (see SciCrunch.org)
SCFAs: short‐chain fatty acid

## 1 Introduction

The gut microbiome comprises a complex community of ~100 trillion microorganisms residing in the digestive tract, > 80% of which belong to species of the *Firmicutes* and *Bacteroidetes* phyla, along with numerous other groups including *Verrucomicobia, Proteobacteria* and *Tenericutes* (Nagpal et al. 2018; Huttenhower et al. 2012). A diverse gut microbiome plays a crucial role in supporting host metabolism by providing metabolic functions not encoded within the host genome. Different microbial species break down complex dietary components such as fibre into short‐chain fatty acids (SCFAs) which serve as energy sources and regulate glucose and lipid metabolism. Greater microbial diversity also enhances metabolic flexibility, improving nutrient extraction and contributing to the synthesis of essential compounds such as vitamins. In addition, the gut microbiome helps maintain gut barrier integrity and limit inflammation, which are closely linked to metabolic health (Cryan et al. 2019; Rusch et al. 2023).

Environmental and genetic factors can influence the gut microbiome, impacting the balance of different phyla and ratios of beneficial and harmful bacteria (Huang et al. 2015; Rusch et al. 2023). These imbalances, known as dysbiosis, can disturb the normal function of the gut microbiome, potentially leading to metabolic and digestive problems, inflammation and impaired immune functioning that over time can contribute to gastrointestinal disorders, such as irritable bowel syndrome and inflammatory bowel disease and obesity (Hrncir 2022; Zhao and Zou 2023). Dysbiosis has also been linked to a range of mental health conditions, including autism, schizophrenia, anxiety and depression. For example, one systematic review and meta‐analysis has reported shared microbiome alterations across multiple psychiatric disorders, including depletion of anti‐inflammatory, short‐chain fatty acid‐producing bacteria and enrichment of potentially pro‐inflammatory taxa, supporting a role for immune and microbial metabolite pathways in the gut–brain axis (Nikolova et al. 2021; Querdasi et al. 2023). A recent study further identified links between alterations in key bacterial genera, including increased *Lactobacillus* and *Escherichia*, with increased risk of neurodevelopmental disorders such as autism (Borrego‐Ruiz and Borrego 2024). In early childhood, multigenerational adversity has also been associated with altered gut microbiome composition and poorer socio‐emotional functioning, providing evidence that social adversity may shape microbiome–brain development during sensitive developmental windows (Querdasi et al. 2023). Together, this highlights the key bidirectional communication network between the brain and the gut microbiome commonly referred to as the gut–brain axis, as well as immune and endocrine systems (Cryan et al. 2020).

The gut microbiome undergoes rapid changes during the postnatal and early life period that starts to stabilise and resemble more an adult‐like composition by early childhood. Early development of the gut microbiome is influenced by numerous factors such as diet and environmental exposures and, in humans, the mode of delivery (Ronan et al. 2021; Frese and Mills 2015; Tamburini et al. 2016). This establishment of the microbiome is crucial for the developing immune and neuronal systems (Ronan et al. 2021). Disruptions to the gut microbiome in early life can increase the risk of various health issues later in life, including inflammatory disorders, autoimmune diseases, neurological disorders and obesity; as such, early life can be considered a vulnerable window (Yatsunenko et al. 2012).

Stress can have a strong influence on the gut microbiome (Cryan et al. 2020). The hypothalamic–pituitary–adrenal (HPA) axis is a key neuroendocrine system that regulates the body's response to stress which, when activated, leads to the release of stress hormones like cortisol (corticosterone in rodents). It is thought that early disturbances to the gut microbiome composition in response to stress may impact brain development thereby leading to increased risk of mental health problems in later life (Beurel and Nemeroff 2024; Callaghan et al. 2020). A recent cross‐species systematic review on the influence of prenatal maternal stress on the offspring gut microbiome including studies from rodents and humans found differences in beta diversity and specific microbial taxa, but not alpha diversity (Graf et al. 2025). The study found results were impacted by variation in study design and operationalization and timing of prenatal stress. There were also variations in microbiome analysis methods, though none had used Nanopore 16S full length Amplicon sequencing that has the potential to offer a more powerful approach for studying microbial diversity (Szoboszlay et al. 2023). There were further effects from the timing of infant microbiome sampling; indeed, many of the included studies showed differences dependent on the age the offspring were when the microbiome was assayed.

Studies in rodents have also revealed that stress during early postnatal life can impact the gut microbiome with effects that persist into adulthood (Jašarević et al. 2015). For example, postnatal stress using the Limited bedding and nesting (LBN) paradigm has been shown to alter gut microbiome composition at weaning, including reduced beneficial bacterial populations and sex‐specific microbial changes alongside increased corticosterone and intestinal permeability (Moussaoui et al. 2017). LBN stress has also been associated with impaired gut barrier integrity, long‐term visceral hypersensitivity and increased anxiety‐like behaviours, suggesting postnatal stress‐induced microbiome changes may contribute to persistent gut–brain axis dysfunction and later vulnerability to stress‐related disorders (Prusator and Greenwood‐Van Meerveld 2015; Holschneider et al. 2016; Guo et al. 2015; Moussaoui et al. 2016). Rodent studies using prenatal maternal restraint stress have also demonstrated significant effects on offspring gut microbiome with reduced microbial diversity, altered abundance of Lactobacillus and Bacteroides species, and long‐term changes in stress responsivity, anxiety‐like behaviour and metabolic function in offspring, suggesting that stress during gestation may programme the gut microbiome (Golubeva et al. 2015; Jašarević et al. 2015).

Here, we aimed to explore further the role of stress exposure timing and sex on microbiome changes. To understand possible mechanisms linking early‐life stress (ELS) to possible microbiome alterations, we treated postnatal offspring with different levels of dexamethasone, a cortisol analogue, and then subsequently tested the effects of exposure to prenatal stress (using maternal restraint) and postnatal stress (using LBN) on the microbiome in later life using Nanopore sequencing. Given that the microbiome undergoes rapid and critical development in infancy, we hypothesised that stress at different early stages might establish different trajectories in the composition and function of the microbiome.

## 2 Methods

### 2.1 Animal Models

**FIGURE 1:**
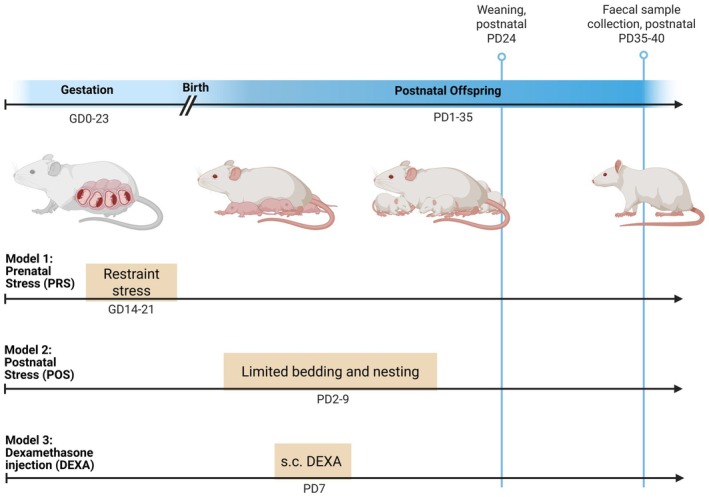
Timeline overview of the three early life stress (ELS) models used in this study. Summary figure outlining the timeline of the study starting prenatally, starting at conception (GD0) and continuing through birth and postnatally into adolescence with weaning at PD24. Model 1. Prenatal Stress (PRS) through restrain stress during GD14‐21, with controls undisturbed. Model 2 Postnatal stress (POS) through limited bedding and nesting material during PD2‐9, with controls having access to normal bedding and nesting material. Model 3 dexamethasone exposure (DEXA), through a subcutaneous (s.c.) injection of low dose (0.5 mg/kg) or high dose (1 mg/kg) dexamethasone, with controls receiving saline only. Faecal samples were taken at adolescence PD35‐40. GD, gestational day; PD, postnatal day.

All animal experiments were performed in accordance with the Animals in Scientific Procedures Act (ASPA) 1986 and locally approved by the Animal Welfare and Ethical Review Body (AWERB) at the University of Manchester. All animal procedures were conducted under the authority of project licences PPL6794596. Adult Wistar rats (RRID:RGD_737929) were obtained from Charles River Laboratories, UK and housed in the Biological Services Facility at the University of Manchester which is maintained on a 12 h light: dark cycle (07:00–19:00) at a temperature of 21°C ± 2°C and humidity 55% ± 5% with *ad libitum* access to standard rat chow (Special Diet Services, Essex, UK) and water. All animals were housed within two‐level individually ventilated cages (Double‐Decker Cage; Tecniplast, Italy), with up to five animals per cage. Three ELS models were used in this study (Figure 1), performed as detailed below:

- Dexamethasone (DEXA) treatment: Wistar rat pups were subcutaneously administered either a high dose (1 mg/kg of body weight) or low dose (0.5 mg/kg of body weight) of dexamethasone (dexamethasone sodium phosphate, 3.3 mg/mL, Hameln Pharma Ltd) or vehicle control (equivalent volume saline), on PD7 between 08:00 and 10:00. These doses and timing were selected from pilot data from our group and previous data from other groups showing these doses reach the developing brain and produce behavioural deficits relevant to neurodevelopmental disorders (Ferguson et al. 2001; Kim et al. 2013; Yates et al. 2016).
- Prenatal stress (PRS) model: pregnant Wistar rat dams at gestational day (GD) 14 were subjected to restraint stress by placing into plastic transparent rodent restrainers for three periods each day (45 min per session at approximately 10:00, 13:00 and 16:00 to minimise circadian effects) until GD21. Control dams were kept undisturbed in their home cages. This model has been previously reported as a paradigm for inducing prenatal stress in offspring (Zuena et al. 2008).
- Postnatal stress (POS) model: Wistar rat dams and their litters were subjected to limited bedding and nesting stress (LBN). In the control group, the dam was provided with an ample amount of wood shavings (a 4‐cm layer), enabling her to construct nests that served as a secure base and caregiving centre for the litter. In the stress group, the dam and litter were placed in a metal mesh platform raised 2.5 cm above the cage floor with approximately 1.5 cm of wood chip bedding underneath the platform. The dam was given half of one paper towel as nesting material, preventing her from building adequate nests for her pups. Litters were subjected to these conditions from postnatal day (PD) 2 to 9. This model was selected as a described paradigm of ELS that disrupts maternal care and induces long‐lasting alterations in stress responsivity in offspring (Rice et al. 2008). For all models, offspring were weaned at PD24 and group‐housed based (*n* = 5 per cage) on their sex and identified using a tail mark. A minimum of five females and five males (one from each litter cage, selected using a random number generator in excel to generate a number between 1 and 5) in each treatment group from each of the three models were used for microbiome analysis from faecal pellets. For faecal sample collection PD35‐40 rats (Sudakov et al. 2021; Sengupta 2013) were single‐housed for up to 3 h 09:30–12:30 on a single day to enable collection of faecal pellets from the base of the cage in the morning. No sample size calculation was performed and the number of subjects used for the study was determined based on previous studies on adult outcomes. All animals were weighed on PD35 and found to have no significant differences with females 120–150 g and males ranging from 140 to 175 g.

### 2.2 Corticosteroid Quantification

Corticosterone concentration was quantified from blood serum taken from tails at PD35 using the Corticosterone ELISA Kit (ENZO, RRID:AB_2307314) according to the manufacturer's instructions and using a 1:40 dilution. The optical density of the plate was read at 405 and 570 nm wavelengths using the Synergy HT microplate reader (Biotex; RRID:SCR_020536).

### 2.3 Microbial DNA Extraction and Sequencing

Collected faecal pellets were processed for microbial DNA using the Microbiome DNA Isolation Kit (Norgen Biotek, Thorold, Canada RRID:SCR_024654) according to the manufacturer's instructions following the Faecal DNA Collection & Preservation Mini Tubes protocol. All DNA samples were quantified using the Qubit dsDNA HS Quantitation kit (Thermo‐Fisher, Loughborough, UK RRID:SCR_018037). Extracted DNA was sequenced using Nanopore sequencing technology. Briefly, library preparation was performed on 10 ng extracted DNA using the 16S Barcoding Kit 24 V14 (Oxford Nanopore Technologies (ONT), Oxford, UK, SQK‐16S114.24) as per the manufacturers' guidelines. This kit allows multiplexing of up to 24 samples to minimise cross‐plate variability and owing to the lack of amplification required using Nanopore technology. We did not use a positive control and while we did use a negative control for the 16S amplification and saw no bands, we did not sequence the negative. 100 fg of the final prepared library was then loaded onto a GridION Flow Cell (ONT, Oxford, UK. RRID:SCR_017986) and sequenced on the ONT GridION Mk1 (RRID:SCR_017986) following the default settings on the 16S workflow within the MinKNOW software (ONT, Oxford, UK. RRID:SCR_003756). The sequencing was performed for 6 h and the FASTQ reads were used for bioinformatics analysis.

### 2.4 Data Analysis

Raw FASTQ files generated on the GridION were basecalled and demultiplexed using standard ONT workflows (MiniKNOW RRID:SCR_003756 and Guppy Basecaller RRID:SCR_023196.). Reads were quality filtered to retain sequences with a mean Q‐score ≥ 10 and lengths between approximately 1300–1700 bp, corresponding to the full‐length 16S rRNA gene. Taxonomic assignment was performed using Emu with the SILVA 138.1 reference database, producing per‐sample read counts at species and genus levels. Reads that could not be confidently classified were labelled as Unassigned and were retained in total read counts when computing relative abundances.

Count tables were imported into a reproducible analysis environment (R and Python), with downstream analyses performed using compositional aware workflows, and converted to relative abundances at genus level for downstream analyses. All compositional analyses retained unclassified reads within the total count denominator to preserve the proportional structure of the data.

#### 2.4.1 Alpha Diversity

Alpha diversity was assessed at genus level using three standard metrics: (i) Observed genera richness; (ii) Shannon diversity; (iii) Simpson diversity. Richness was calculated for genera with relative abundance ≥ 0.0001 to reduce noise from extremely low‐abundance taxa. Diversity indices were computed using the vegan and phyloseq R packages. To ensure robust handling of compositional microbiome data, primary analyses were processed via the EMU pipeline, which incorporates stringent taxonomic assignment and quality control, followed by compositional‐aware transformations. To evaluate potential sequencing depth effects, sensitivity analyses were performed using rarefaction to a common read depth (Figure S1), the results were qualitatively unchanges, indicating cohort and treatment effects were not driven by differences in sequencing depths. However, primary analyses were conducted on the full relative‐abundance dataset.

For each ELS model (PRS, POS, DEXA), alpha diversity was analysed using general linear models with sex, treatment and sex*treatment interaction included as fixed factors. For the DEXA cohort (where sequencing was performed across 2 plates), sequencing plate was included as an additional covariate. *p*‐values were corrected for multiple testing across alpha metrics within each model using the Benjamini‐Hochberg false discovery rate (FDR).

#### 2.4.2 Beta Diversity

Prior to multivariate analyses, genus‐level relative abundance data were transformed using centred‐log‐ratio (CLR) transformation to account for composition constraints. Community compositional differences were evaluated using Aitchison distances, computed as Euclidean distances on centred CLR‐transformed genus‐level relative abundances. Ordination was performed using principal component analysis (PCoA) on CLR‐transformed data, with samples visualised according to treatment/dose and sex. Group differences in microbiome composition were tested using PERMANOVA (999 permutations) with the same model structures used as alpha diversity. To distinguish differences in centroid location from differences within‐group dispersion, homogeneity of multivariate dispersion was evaluated using PERMDISP.

#### 2.4.3 Beta Diversity Driver

To identify taxa contributing most strongly to community‐level differences, beta‐diversity driver analyses were performed by correlating genus‐level relative abundances with the first two principal coordinate axes derived from the CLR‐transformed Aitchison distance matrix. Spearman correlation coefficients were computed between each genus and PCoA axes and taxa were ranked according to the magnitude of their association with ordination structure. Genera with the strongest correlations were interpreted as candidate contributors to beta‐diversity patterns. These analyses were performed separately for each ELS model, and results were used for interpretation of multivariate structure rather than formal hypothesis testing.

#### 2.4.4 Differential Abundance

Differential abundance analyses were conducted at genus level using non‐parametric tests on relative abundances: (i) For PRS and POS, stress and control groups were compared using Wilcoxon rank‐sum tests. (ii) For DEXA, differences across untreated, low‐dose and high‐dose groups were tested using Kruskal–Wallis tests, followed by pairwise Wilcoxon tests for exploratory contrasts. Effect sizes were defined as differences in group medians, and Benjamini–Hochberg FDR correction was applied across genera within each model. Genera showing nominal or near‐significant associations were visualised using volcano plots and heatmaps of the most abundant taxa.

#### 2.4.5 Machine Learning Analysis

To capture higher‐order computational structure, Random Forest classification models were applied to genus‐level relative abundance data. Models were trained to classify treatment/dose groups within each ELS paradigm using stratified k‐fold cross‐validation. Classification performance was assessed using confusion matrices and predicted class probabilities.

To evaluate the influence of sex on predictive structure, models were trained on combined datasets and on sex‐stratified subsets. To assess cross‐model similarity, PRS and POS samples were projected into models trained on DEXA data, and predicted class probabilities were compared across groups. Feature importance scores were extracted from Random Forest models to identify taxa contributing most strongly to classification. These were compared qualitatively with taxa identified in beta‐diversity driver analyses to assess concordance between supervised and unsupervised approaches.

#### 2.4.6 Exploratory Compositional Analysis

To further investigate compositional differences not captured by the primary diversity analyses, exploratory differential abundance testing was performed using the ALDEx2 framework. Genus‐level count tables were analysed using ALDEx2, which models compositional data under a centred log‐ratio (CLR) transformation and accounts for sampling variability through Monte Carlo sampling from Dirichlet distributions. For each comparison, 128 Monte Carlo instances were generated to estimate the expected CLR values and associated variance.

Analyses were conducted separately for each ELS model (PRS, POS, DEXA) and additionally stratified by sex to evaluate potential sex‐dependent compositional effects: (i) For PRS and POS, two‐group comparisons between stress and control animals were performed using Welch's *t*‐test within the ALDEx2 framework. (ii) For DEXA, multi‐group comparisons across untreated, low‐dose and high‐dose conditions were performed using the Kruskal–Wallis test implemented in ALDEx2. For each genus, expected effect sizes (difference in CLR abundance between groups) and associated *p*‐values were calculated. Multiple testing correction was applied across genera within each comparison using the Benjamini‐Hochberg false discovery rate (FDR).

Results were summarised for cohort‐wide comparisons and sex‐stratified analyses within each ELS model and visualised using volcano plots displaying effect size versus –log_10_(FDR‐adjusted *p*‐value), with genera exceeding nominal or FDR‐adjusted thresholds highlighted for interpretation. Given the modest sample size and compositional nature of the data, these analyses were considered exploratory and were not used to define primary outcomes but rather to support interpretation of multivariate community‐level findings.

### 2.5 Software and Data Repository

All analyses were conducted in Python and R. Diversity indices were computed using standard implementations consistent with vegan/phyloseq definitions. Complete analysis scripts, intermediate count tables, are provided in the supporting information at https://dataview.ncbi.nlm.nih.gov/object/PRJNA1492029?reviewer=bfitk1tqed7lfjmja2gj2et2u2.

## 3 Results

### 3.1 Microbiome Sequencing and Taxonomic Profiling

Corticosterone analysis of PRS and POS rats aged 35 days old revealed that while PRS showed no differences between stress and controls, both male and female POS stress rats had elevated Corticosterone compared to controls (Figure S2).

Across all samples, full‐length 16S sequencing yielded high‐quality taxonomic profiles comprising 84 samples and 132 bacterial genera following quality control and filtering. The majority of reads were successfully assigned to known bacterial taxa using the SILVA reference database, with unclassified reads retained in compositional denominators to preserve relative abundance structure.

All three ELS cohorts—prenatal stress (PRS), postnatal stress (POS) and dexamethasone exposure (DEXA) showed broadly similar overall microbiome structures dominated by genera commonly observed in murine gut microbiota. Genus‐level abundance distributions were consistent across sequencing runs, and sequencing depth sensitivity analyses confirmed that diversity metrics and ordination structure were stable across read depth thresholds (Figure S3).

### 3.2 Cohort‐Wide Microbiome Structure

**FIGURE 2:**
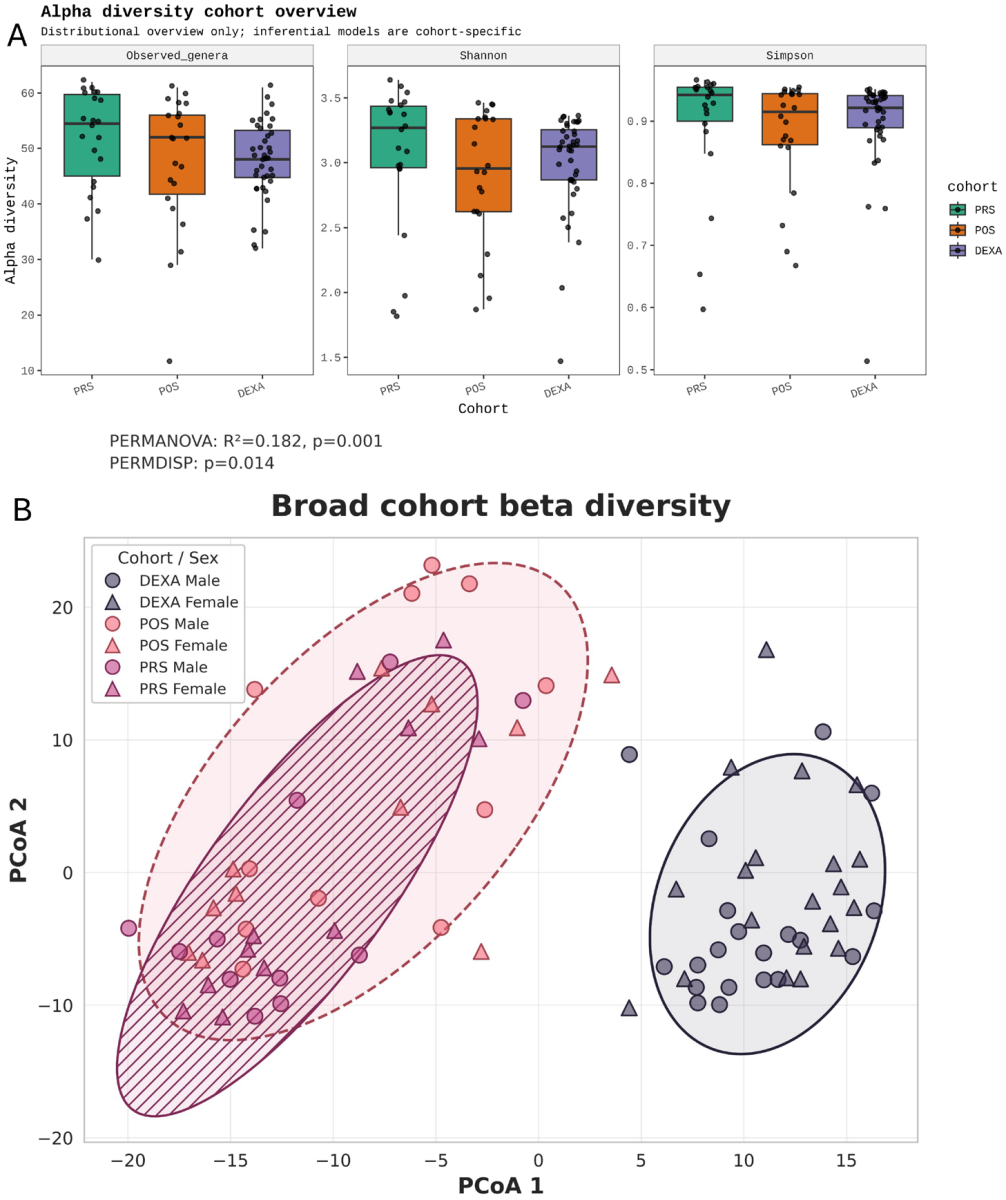
Cohort‐level alpha and beta diversity overview. (A) Alpha Diversity metrics across cohorts (PRS, POS, DEXA), including Observed Genera, Shannon diversity and Simpson diversity. Points represent individual samples; boxplots show median and interquartile range (IQR), with whiskers extending to 1.5× IQR. (B) Principal coordinate analysis (PCoA) of beta diversity showing overall microbiome compositional differences between cohorts. Points represent individual samples, coloured by cohort and stratified by sex (circles = male, triangles = female). Ellipses indicate cohort‐level dispersion (95% confidence). A clear separation is observed between DEXA and the early‐life stress cohorts (PRS and POS), with partial overlap between PRS and POS. PEMANOVA indicates a significant effect of cohort on microbial community composition (*R* ^2^ = 0.182, *p* = 0.001). Permutational analysis of multivariate dispersions (PERMPDISP) was also significant (*p* = 0.014), indicating modest differences in within‐group dispersion. *n* = 5–7/sex/group.

To assess global differences in microbial diversity across ELS paradigms, alpha and beta diversity across the combined PRS, POS and DEXA cohorts was examined (Figure 2). Alpha‐diversity differed modestly across cohorts, with PRS samples exhibiting the highest average richness and diversity compared to POS and DEXA (Figure 2A). Within‐group variability across all cohorts was substantial.

In contrast, beta diversity analysis revealed clear compositional separation between cohorts. PCoA of Aitchison distance demonstrated distinct clustering, particularly separating DEXA samples from PRS and POS (Figure 2B). PERMANOVA confirmed significant effect of cohort on community composition (*R* ^2^ = 0.182, *p* = 0.001), indicating that ELS model type explains a meaningful proportion of microbiome variance. However, PERMDISP was also significant (*p* = 0.014), suggesting that differences in dispersion contribute partially to the observed separation. Together, these results indicate that within‐samples diversity varies only modestly, between‐cohort compositional structure differs substantially across ELS paradigms.

These findings indicate that cohort level differences (PRS, POS, DEXA) represent dominant sources of variation in microbiome composition, likely reflecting temporal, experimental, or housing‐related factors. Consequently, all further analyses were conducted within cohorts, rather than direct cross‐cohort comparisons. This approach allows for the detection of stress‐related effects while accounting for underlying cohort structure and allows for the identification of shared directional effects across independent experimental cohorts without confounding by between‐cohort variability.

### 3.3 DEXA Induces Sex‐Specific Shifts in Microbial Community Composition

**FIGURE 3:**
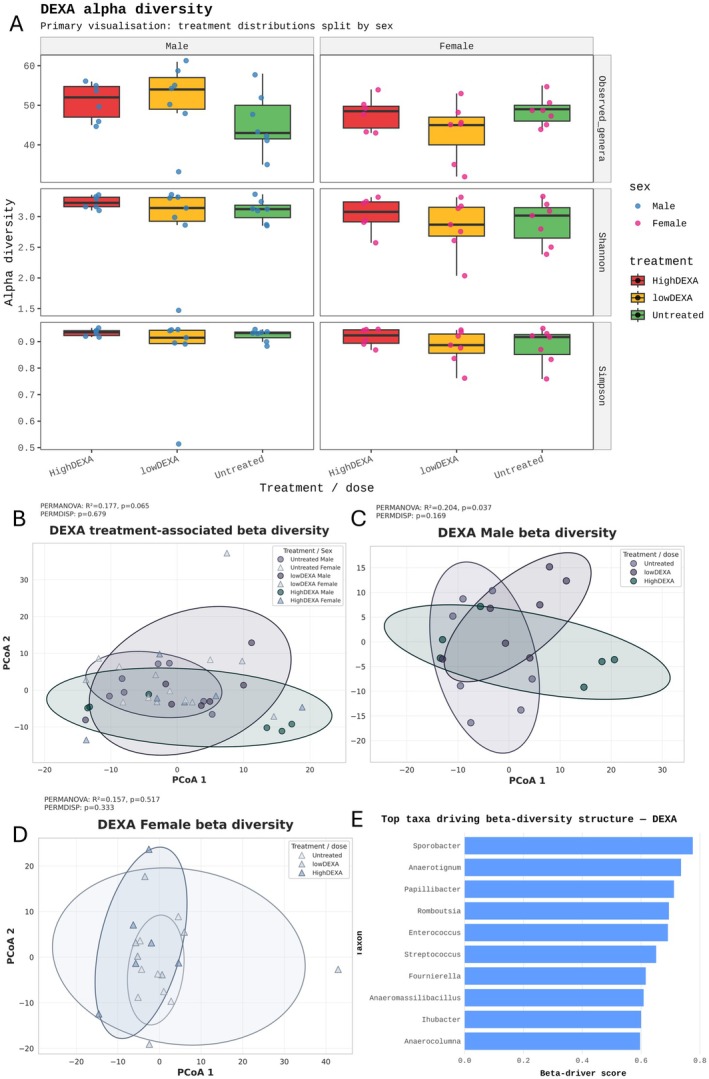
Sex‐stratified dexamethasone cohort alpha and beta diversity analysis with beta drivers. (A) Alpha Diversity across DEXA treatment groups (HighDexa, LowDexa, Untreated), stratified by sex (male and female), shown for Observed genera, Shannon diversity and Simpson diversity. Points represent individual samples; boxplots indicate median and interquartile range (IQR), with whiskers extending to 1.5× IQR. This provides a descriptive overview of within‐sample diversity across treatment conditions within each sex. (B) Principal Coordinate analysis (PCoA) of beta diversity across all DEXA samples, coloured by treatment and stratified by sex (circles = male, triangles = female). Ellipses represent 95% confidence intervals for each group. No significant effect of treatment was observed at the cohort level (PERMANOVA: *R* ^2^ = 0.177, *p* = 0.065) with no evidence of dispersion (PERMDISP: *P* = 0.0679). (C) Male only beta diversity analysis within the DEXA cohort. A significant effect of treatment was detected (PERMANOVA: *R* ^2^ = 0.204, *p* = 0.037), with no significant difference in dispersion (PERMDISP: *P* = 0.679). (C) Male‐only beta diversity within the DECA cohort. A significant effect of treatment was detected (PERMANOVA: *R* ^2^ =0.204, *p* = 0.037), with no significant difference in dispersion (PERMDISP *p* = 0.169), indicating a treatment‐associated shift in community composition in males. (D) Female‐only beta diversity analysis within the DEXA cohort. No significant treatment effect was observed (PERMANOVA: *R* ^2^ = 0.157, *p* = 0.517), and dispersion was not significantly different (PERMDISP *p* = 0.33). (E) Top bacterial genera contributing to beta diversity structure within the DEXA cohort, ranked by their contribution to ordination separation. These taxa represent the primary divers of the compositional differences observed in the PCoA analysis. *n* = 6–7/sex/group.

Further examining cohort‐specific trends requires stratifying sexually dimorphic trends and treatment dose differences (Figure 3). Alpha diversity analysis revealed no strong treatment‐associated differences across observed genera, Shannon or Simpson indices in either sex (Figure 3A). While minor trends were apparent, these did not translate into robust statistical separation.

In contrast, beta‐diversity analyses revealed sex‐specific compositional effects. At the cohort level, DEXA treatment showed a trend toward compositional differences (PERMANOVA *R* ^2^ = 0.177, *p* = 0.065; Figure 3B), although this did not reach statistical significance. When stratified by sex, a significant divergence between males and females is observed. Males, demonstrate a significant treatment trend (PERMANOVA *R* ^2^ = 0.204, *p* = 0.037; Figure 2C) where no such effect was observed in females (PERMANOVA *R* ^2^ = 0.157, *p* = 0.717; Figure 2D).

Importantly, PERMDISP was non‐significant in all cases, indicating that these differences are driven by shifts in community centroid rather than dispersion. Analysis of taxa contributing to beta‐diversity structure identified several genera associated with DEXA‐driven variation (Figure 2E), suggesting that treatment effects are mediated by coordinated multivariate shifts rather than large changes in individual taxa.

### 3.4 Limited Compositional Disruption in PRS Despite Preserved Community Structure

**FIGURE 4:**
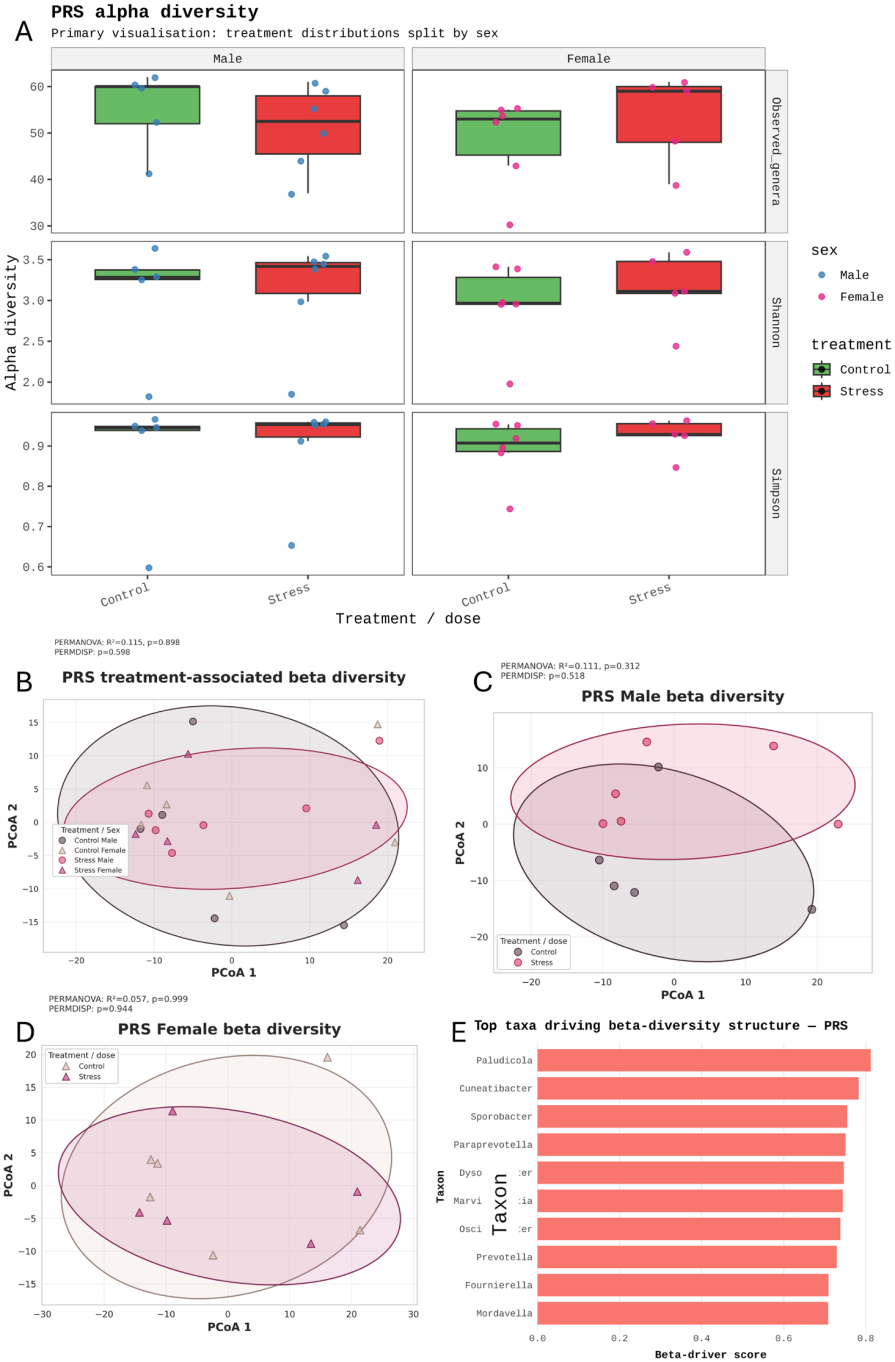
Sex‐stratified alpha and beta diversity analysis within the prenatal stress (PRS) cohort. (A) Alpha diversity across PRS treatment groups (control and stress), stratified by sex (male and female), shown for observed genera, Shannon diversity and Simpson diversity. Points represent individual samples; boxplots indicate median and IQR with whiskers extending to 1.5× IQR. This provides a descriptive overview of within‐sample diversity under PRS conditions within ach sex. (B) Principal coordinate analysis (PCoA) of beta diversity across all PRS samples, coloured by treatment and stratified by sex (Circles = male, triangle = female). Ellipses represent 95% confidence intervals for each group. No significant effect of prenatal stress was observed at the cohort level (PERMANOVA: *R* ^2^ = 0.111, *p* = 0.898) and no differences in dispersion were detected (PERMDISP *p* = 0.598). (C) Male‐only beta diversity analysis within the PRS cohort. No significant treatment‐associated differences in microbial community composition were observed (PERMANOVA *R* ^2^ = 0.111, *p* = 0.312), and dispersion was not significantly different (PERMDISP *p* = 0.518). (D) Female‐only beta diversity analysis within the PRS cohort. No significant effect was PRS was detected (PERMANOVA *R* ^2^ = 0.057, *p* = 0.999), with no evidence of dispersion differences (PERMDISP *p* = 0.944). (E) Top bacterial genera contributing to beta diversity structure within the PRS cohort, ranked by their contribution to ordination separation. These taxa represent the principal contributors to variation in microbiome composition, despite the absence of significant treatment‐associated clustering. *n* = 5–6/sex/group.

In the PRS model, alpha diversity metrics showed minimal differences between stress and control groups across both sexes (Figure 4A). Observed genera, Shannon and Simpson indices were broadly comparable, indicating preserved within‐sample diversity.

Consistent with this, beta diversity analyses revealed no significant treatment‐associated differences at the cohort level (PERMANOVA *R* ^2^ = 0.115, *p* = 0.898; Figure 3B). Stratified analyses in males (Figure 4C) and females (Figure 4D) similarly showed no evidence of compositional separation.

Despite the absence of global compositional shifts, analysis of taxa contributing to ordination structure identified a subset of genera associated with PRS‐related variation (Figure 3E). These findings suggest that PRS induces subtle, distributed changes in microbial composition that are not captured by global diversity metrics.

### 3.5 POS Exhibits Modest but Detectable Compositional Restructure

**FIGURE 5:**
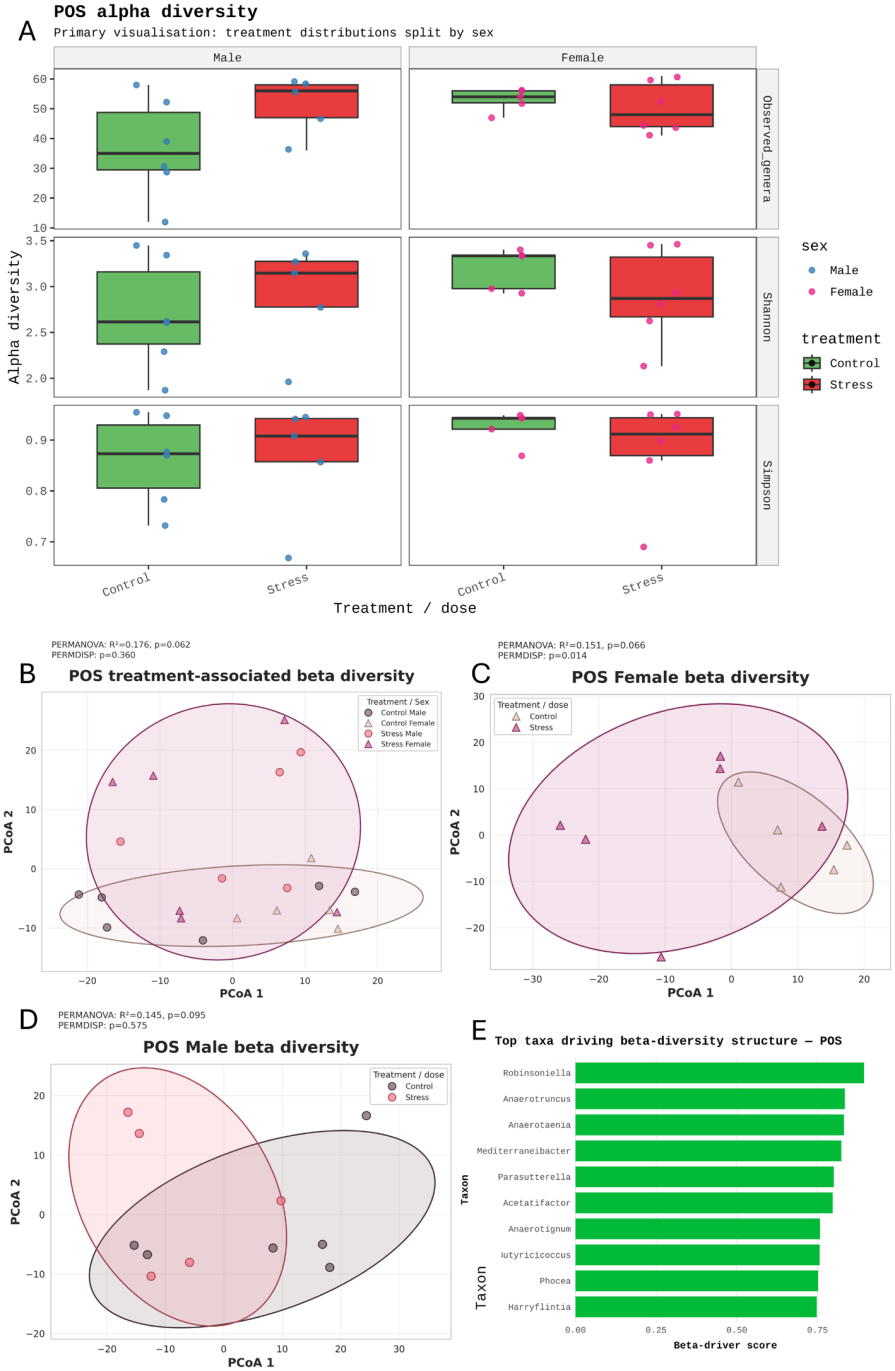
Sex‐stratified alpha and beta diversity analysis within the postnatal stress (POS) cohort. (A) Alpha diversity across POS treatment groups (control and stress), stratified by sex (male and female), shown for observed genera, Shannon diversity and Simpson diversity. Points represent individual samples; boxplots indicate median and IQR, with whisker extending 1.5× IQR. This provides a descriptive overview of within‐sample diversity under postnatal stress conditions within each sex. (B) PCoA of beta diversity across all POS samples, coloured by treatment and stratified by sex. Ellipses represent 95% confidence interval for each group. A trend toward treatment‐associated differences in community composition was observed (PERMANOVA *R* ^2^ = 0.176, *p* = 0.062), with no evidence of differences in dispersion (PERMISP *p* 0.360). (C) Female‐only beta diversity analysis within the POS cohort. A near‐significant trend toward treatment associated compositional differences was observed (PERMANOVA *R* ^2^ = 0.151 *p* = 0.066), with a significant difference in dispersion (PERMDISP *p* = 0.014), indicating that variability within groups may contribute to the observed separation. (D) Male‐only beta diversity analysis within the POS cohort. No significant treatment‐associated differences were detected (PERMANOVA *R* ^2^ = 0.145, *p* = 0.095), and dispersion was not significantly different (PERMDISP *p* = 0.575). (E) Top bacterial genera contributing to beta diversity structure within the POS cohort, ranked by their contribution to ordination separation. These taxa represent the primary drivers of the compositional variation observed in the PCoA analysis. *n* = 5–6/sex/group.

In contrast to PRS, the POS model demonstrated evidence of compositional shifts, particularly in females (Figure 5). Alpha diversity showed modest variation between control and stress groups, with no consistent directional effect across metrics (Figure 5A). However, beta‐diversity analysis revealed trends toward treatment‐associated separation at the cohort level (PERMANOVA *R* ^2^ = 0.176, *p* = 0.062; Figure 5B).

Stratification by sex revealed a clearer signal in females (PERMANOVA *R* ^2^ = 0.151, *p* = 0.066; PERMDISP *p* = 0.014; Figure 5C), whereas males showed weaker and non‐significant differences (PERMANOVA *R* ^2^ = 0.145, *p* = 0.095; Figure 5D). The significant PERMDISP in females suggests that increased within‐group variability, suggesting that dispersion may contribute to the observed pattern. However, these sex‐stratified effects were attenuated following rarefaction, indicting that they are modest and sensitive to subsampling. Genera contributing to beta‐diversity structure in the POS cohort (Figure 5E) overlapped partially with those observed in other models.

### 3.6 Community‐Level Differences Are Not Driven by Large Shifts in Individual Taxa

To determine whether observed compositional differences were driven by specific taxa, we performed differential abundance analyses across all cohorts (Figure 7). Volcano plots revealed effect sizes were generally small and centred near zero across the DEXA, PRS and POS models (Figure 7A–C). No genera remained significant following FDR correction in DEXA and PRS cohorts; however, Parabacteroides (FDR = 0.047) and Tyzzerella (FDR = 0.047) remained significant in the POS cohort. Exploratory compositional analysis using ALDEx2 yielded highly concordant results, identifying the same genera in the POS cohort while confirming the absence of FDR‐significant taxa in PRS and DEXA, supporting the robustness of these findings to compositional modelling approaches.

**FIGURE 6:**
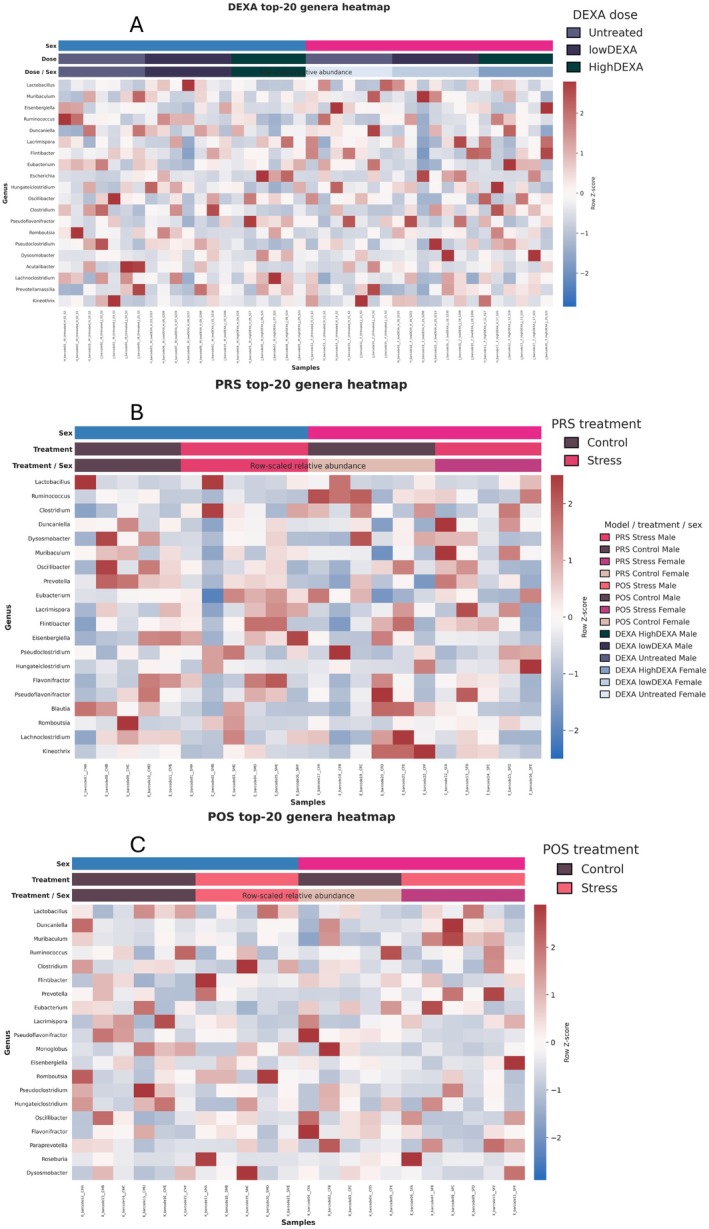
Top 20 genera heatmaps across DEXA, PRS and POS cohorts. (A–C) Heatmaps showing the top 20 most abundant bacterial genera within each cohort: DEXA (A), PRS (B) and POS (C). Genera are displayed as rows and samples as columns, with values representing row‐scaled (*z*‐scored) relative abundance, enabling comparison of within‐genus variation across samples. Samples are annotated by sex (blue = male, pink = female) and treatment condition (DEXA: Untreated, lowDEXA, HighDEXA; PRS/POS: Control, stress). For DEXA, treatment is further stratified by sex in the annotation bar to reflect combined dose‐sex grouping. Colour intensity reflects deviation from the mean abundance of each genus across samples (red = high relative abundance; blue = lower relative abundance), highlighting cohort‐specific and treatment‐associated patterns of microbial variation. Across cohorts, distinct genera exhibit heterogeneous abundance patterns, with some taxa showing clustering by treatment and/or sex, consistent with compositional differences in observed beta diversity analysis. *n* = 5–7/sex/group.

These findings indicate that microbiome differences across the ELS insults are generally not driven uniformly, with DEXA and PRS cohorts appearing to incur coordinated multivariate changes in community structure. While POS structural changes did have more specific genera effects, heatmaps of the top 20 most abundant genera further support this interpretation—showing distributed variation across samples without clear, treatment‐specific clustering (Figure 6).

### 3.7 Machine Learning Reveals Cross‐Cohort Microbial Signatures and Sex‐Dependent Effects

**FIGURE 7:**
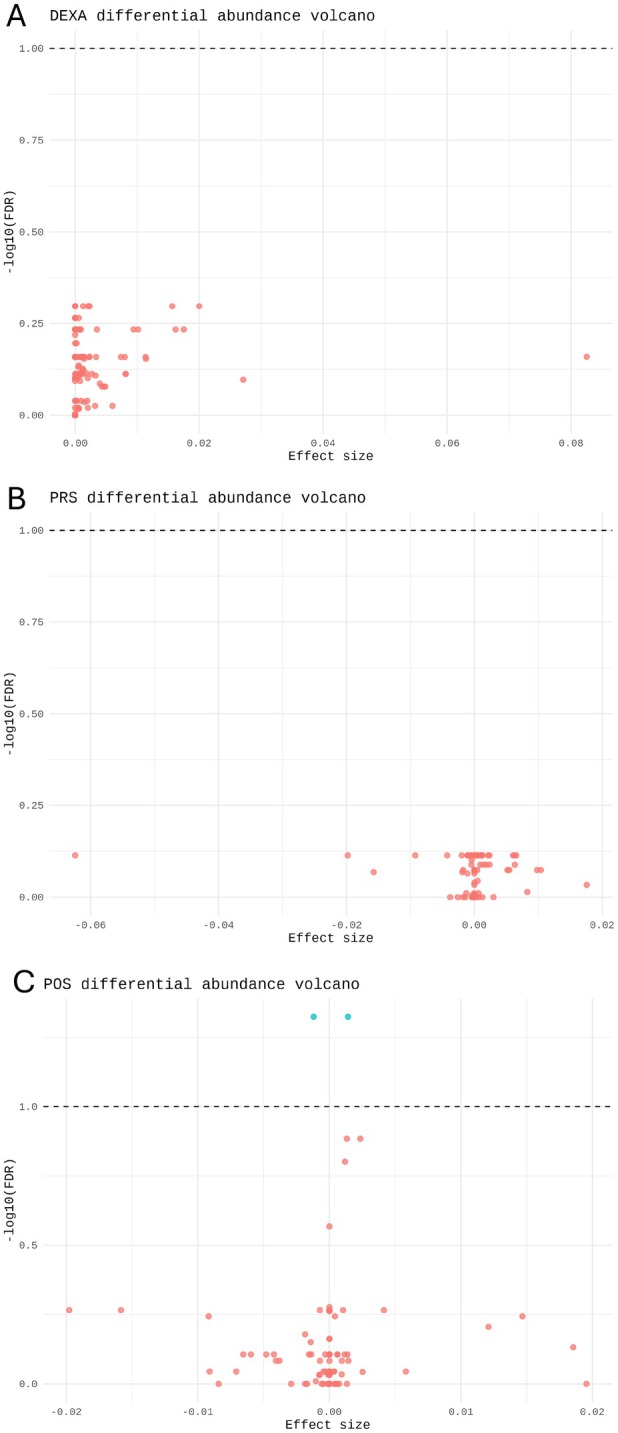
Differential abundance analysis across DEXA, PRS and POS Cohorts. (A–C) Volcano plots showing differential abundance of bacterial genera between treatment groups within each cohort: DEXA (A), PRS (B) and POS (C). Each point represents a genus, plotted by effect size (*x*‐axis) and –log_10_(FDR‐adjusted *p*‐value) (*y*‐axis). The dashed horizontal line indicates the significance threshold (FDR = 0.05). Across all cohorts, no genera passed the multiple testing threshold followed false discovery rate (FDR) correction, indicating an absence of robust differentially abundant taxa at the genus level. Effect sizes were generally small and centred near zero, suggesting that treatment‐associated differences are modest and not driven by large shifts in individual taxa. In the POS cohort, two genera survived FDR correction remaining significant, Parabacteroides and Tyzzerella. Similarly, DEXA and PRS cohorts showed limited evidence of differential abundance, consistent with broader compositional analysis. *n* = 5–7/sex/group.

To capture higher‐order compositional patterns, we applied Random Forest classification to genus‐level profiles (Figure 7). Classification performance in the combined dataset was modest (Figure 7A), with improved discrimination observed in sex‐stratified models (Figure 7B,C), indicating that sex‐specific microbial signatures enhance predictive power.

Projection of PRS and POS samples into DEXA‐trained models revealed partial alignment with DEXA‐derived microbial states (Figure 7D–F), suggesting shared underlying community features across ELS paradigms. These patterns were further modulated by sex, with distinct prediction distributions observed between males and females (Figure 7G–I).

Analysis of feature importance identified key taxa contributing to classification (Figure 7J–L). Notably, several of these taxa overlapped with those identified as beta‐diversity drivers, indicating concordance between multivariate ordination and machine learning approaches.

## 4 Discussion

Early‐life stress (ELS) is widely recognised as a major determinant of long‐term physiological and neurobiological outcomes (O'Mahony et al. 2009), however, its effect on hut microbiome structure remains inconsistently defined across experimental models. In particular, it remains unclear whether different ELS paradigms induce convergent microbial responses, or whether microbiome alterations are highly model‐specific and context dependant. In this study, three commonly used murine ELS models—prenatal restraint stress (PRS), postnatal stress (POS) and dexamethasone exposure (DEXA)—were analysed within a unified framework integrating alpha diversity, compositional beta diversity and machine learning approaches.

**FIGURE 8:**
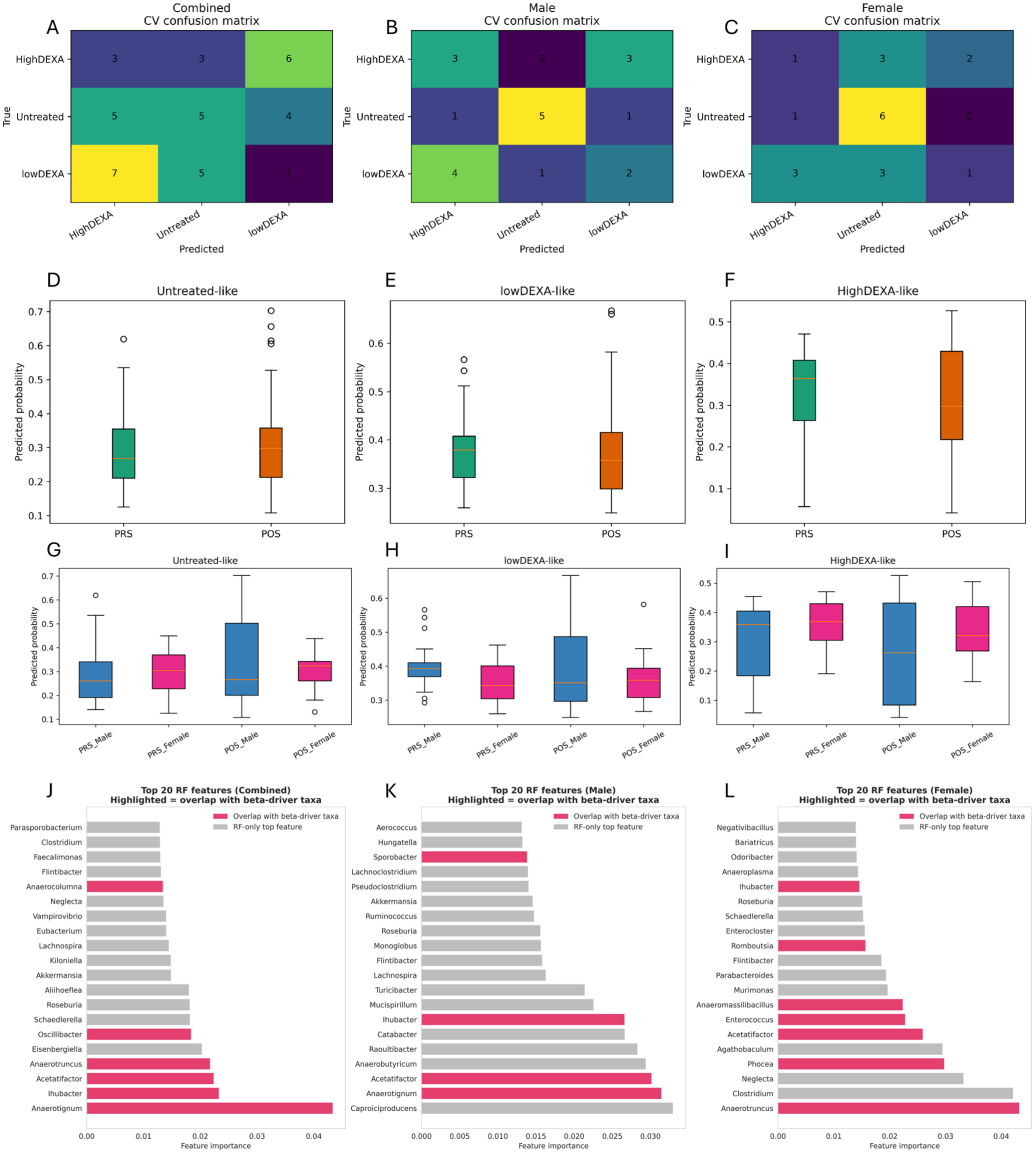
Machine learning classification performance and feature importance across cohorts. (A–C) Cross‐validated confusion matrices for Random Forest classification of treatment groups (HighDEXA, lowDEXA, Untreated) using microbiome profiles in the combined cohort (A), males (B) and females (C). Rows represent true labels and columns predicted labels. Classification performance varies across strata, with improved discrimination observed in sex‐stratified models relative to the combined cohort. (D–F) Predicted class probabilities for PRS and POS samples projected into the DEXA‐trained model, shown for Untreated‐like (D), lowDEXA‐like (E) and HighDEXA‐like (F) classifications. Distributions indicate partial overlap between cohorts, suggesting shared microbial signatures across experimental paradigms. (G–I) Sex‐stratified predicted probabilities for PRS and POS samples (male and female) across the same DEXA‐derived classification axes (untreated‐like, lowDEXA‐like, HighDEXA‐like). Sex‐specific differences in prediction distributions highlight potential interaction effects between sex and treatment‐associated microbial structure. (J–L) Top 20 Random Forest features (genera) contributing to classification in the combined (J), male (K) and female (L) models. Genera highlighted in colour represent taxa that overlap with those identified as beta‐diversity drivers, indicating concordance between multivariate community structure and machine learning feature importance. Non‐highlighted taxa represent features uniquely identified by the Random Forest model. *n* = 5–7/sex/group.

Overall, the data presented here suggest that microbiome alterations associated with the ELS are subtle and model‐dependent and are primarily reflected at the level of community structure rather than large‐scale changes in individual taxa. While differential abundance analyses identified a small number of genera associated with the POS model, the majority of observed effects were distributed across microbial communities, indicating that ELS does not induce a classical dysbiosis phenotype but instead results in coordinated shifts in microbial composition (Figure 8).

### 4.1 Multivariate Restructuring Predominates Over Discrete Taxonomic Shifts

A consistent pattern observed across all models was the divergence between multivariate and univariate analytical approaches. PERMANOVA analysis revealed trend‐level compositional differences in both POS (*R* ^2^ = 0.176, *p* = 0.062) and DEXA (*R* ^2^ = 0.177, *p* = 0.065), with a significant treatment associated effect observed in DEXA males (*R* ^2^ = 0.204, *p* = 0.037). In contrast, genus‐level differential abundance testing identified only two taxa that remained significant following FDR correction, both within the POS model, with no significant taxa detected in PRS or DEXA cohorts.

This divergence suggests that ELS‐associated microbiome changes are not driven by expansion or depletion of individual taxa but instead reflect distributed ecological restructuring across microbial communities. Such restructuring is consistent with modest shifts in relative abundance across multiple taxa, each contributing incrementally to overall community composition, rather than discrete taxonomic perturbations. This interpretation is further supported by the absence of strong clustering in heatmap visualisations, which show distributed variation across samples without clear treatment‐specific signatures.

### 4.2 Model‐Specific Microbiome Responses to Early‐Life Stress

#### 4.2.1 DEXA Induces Sex‐Dependent Compositional Restructuring

Among three models investigated, dexamethasone exposure produced the most consistent evidence of microbiome restructuring, particularly following sex stratification. While the overall cohort‐level effect remained modest and did not reach statistical significance, analyses of male samples revealed a significant treatment‐associated shift in community composition. The absence of a corresponding effect in females indicates a clear sex‐dependent response to glucocorticoid exposure. Importantly, these compositional changes were not accompanied by significant differential abundance of individual taxa, suggesting that DEXA induces coordinated shifts in microbial community organisation rather than discrete taxonomic changes. This interpretation is strengthened by the observation that taxa contributing most strongly to beta‐diversity structure also ranked highly in Random Forest classification models, indicating that these features represent reproducible aspects of the microbial response. We are not aware of any other rodent studies that have tested effects of postnatal DEXA treatment. Interestingly, one study of prenatal treatment in Wistar rats led to long‐term, sex‐dependent alterations in the gut microbiome with pronounced effects in beta‐diversity seen in adult female offspring, with changes seen in overall community structure rather than large shifts in individual taxa (Lu et al. 2024). This opposite effect of female effects to males might reflect the importance of different developmental times. Another study in male mice found that DEXA reduced alpha diversity and increased the ratio of Firmicutes/Bacteroidota; however, this model used 25× higher doses of DEXA than here that were intraperitoneally injected over 4 weeks in adulthood (Qiu et al. 2023), perhaps reflecting higher impacts of higher chronic doses.

These findings suggest that glucocorticoid exposure may be one pathway through which ELS shapes the gut microbiome. Rather than targeting specific bacteria, DEXA may alter the host intestinal environment through glucocorticoid receptor signalling, immune regulation, epithelial barrier function and metabolism, producing microbiome community‐level shifts without strong differential abundance of specific individual taxa (Tena‐Garitaonaindia et al. 2022; Huang et al. 2015). The male‐specific effect may also reflect interactions between glucocorticoid exposure and sex‐dependent immune, hormonal and microbial development, consistent with evidence that sex hormones and microbiota interact during early life (Markle et al. 2013; He et al. 2021). Together, this supports a model in which early glucocorticoid exposure indirectly programmes the microbiome through host physiology, resulting in sex‐dependent compositional restructuring.

#### 4.2.2 PRS Exhibits Minimal Detectable Microbiome Disruption

In contrast to DEXA, prenatal stress produced minimal detectable changes in microbiome composition. Alpha diversity metrics remained stable across treatment groups, and beta‐diversity analyses showed no evidence of compositional separation at either cohort or sex‐stratified levels. While exploratory analyses identified taxa associated with ordination structure, these findings were not supported by global compositional shifts or differential abundance results and should therefore be interpreted with some caution. This is broadly consistent with recent synthesis showing that prenatal stress effects on offspring microbiota are often inconsistent, with limited evidence for altered alpha diversity and effects depending on species, stress timing, sampling age and analytical approach (Graf et al. 2025). While some rodent studies report sex‐ or time‐specific microbial alterations following prenatal stress, these effects are not always reflected in broad community‐level restructuring (Jašarević et al. 2017). Taken together, these results suggest that PRS‐induced physiological effects may occur independently of large‐scale microbiome restructuring or may involve transient or subtle alterations that are not captured within the resolution of the present 16S‐based analysis. This is consistent with the possibility that prenatal stress effects are mediated primarily through host developmental pathways, with limited downstream impact on microbial community structure during adolescence.

#### 4.2.3 POS Induces Heterogeneous Community Restructuring With Targeted Taxonomic Effects

The POS model exhibited an intermediate phenotype, characterised by modest compositional shifts alongside increased community variability. While PERMANOVA indicated only a trend‐level effect at the cohort level, significant differences in dispersion were observed in females, suggesting that postnatal stress induces heterogeneous microbiome responses rather than a uniform directional change. This pattern may reflect the nature of the LBN model, in which stress exposure is mediated through the disruption of the early caregiving environment and dam–pup interactions that can influence offspring HPA‐axis development, gut barrier maturation, immune signalling, feeding patterns and maternal microbial transmission, all of which may affect microbiome assembly (Moussaoui et al. 2016, 2017). Importantly, because maternal behavioural responses to limited bedding can vary across dams and litters, downstream microbial effects may also be heterogeneous, consistent with increased dispersion rather than a single uniform community shift.

Notably, POS was the only model in which individual taxa remained significant following FDR correction. Specifically, *Parabacteroides* was increased and *Tysserella* decreased in stress‐exposed animals. These taxa have previously been associated with host metabolic and inflammatory processes, and their opposing directional changes may reflect selective alterations within specific ecological niches.

The combination of increased dispersion and targeted taxonomic changes may suggest greater variability in microbial community structure under postnatal stress conditions. However, the lack of consistency across models and attenuation of this effect following rarefaction indicate that these differences are modest and context‐dependent rather than indicative of a robust or generalised destabilisation phenotype. This interpretation is consistent with broader patterns of distributed compositional shifts observed across the dataset.

#### 4.2.4 Sex as a Key Modifier of Microbiome Responses

Sex emerged as an important modifier of microbiome responses to ELS, although this effect was not uniform across models. Within the limits of the sample size, DEXA‐associated compositional changes were confined to males, whereas POS‐related increases in dispersion were more evident in females. PRS, in contrast, did not exhibit detectable sex‐specific effects. This suggests that sex‐dependent host factors may influence how microbial communities respond to environmental perturbations. Sex hormones such as oestrogens and androgens are known to shape the gut microbiome by altering immune responses, gut physiology and microbial metabolism, while the microbiome itself can regulate circulating hormone levels (Markle et al. 2013). Furthermore, fluctuations in these hormones, such as those occurring across the oestrous cycle, can further influence microbiome composition (Jašarević et al. 2016). Importantly, these results also indicate that pooling male and female samples may obscure biologically meaningful effects, particularly in models such as DEXA where responses are strongly sex‐dependent.

### 4.3 Convergence of Beta‐Diversity Drivers and Machine Learning Features

A notable finding of this study was the partial overlap between taxa identified as a major contributor to beta‐diversity structure and those ranked as important features in Random Forest classification models. Several genera, including *Robinsoniella*, *Anaerotruncus* and *Sporobacter*, were consistently identified across both analytical approaches.

While this overlap was not complete, it suggests that microbiome responses to ELS are organised along reproducible ecological axes, rather than representing stochastic variation. The fact that both unsupervised (ordination‐based) and supervised (classification‐based) methods converge on similar taxa supports the interpretation that these genera represent biologically meaningful contributors to community restructuring.

Classification performance was modest in combined datasets but improved following sex stratification, further supporting the presence of sex‐dependant microbial signatures. Together, these findings indicate that ELS‐associated microbiome changes are structured and reproducible, even when individual effect sizes are small.

### 4.4 Limitations

Several limitations should be considered when interpreting these findings. First, samples sizes within sex‐stratified analyses were modest, limiting statistical power to detect subtle effects. Second, all analyses were conducted at a single adolescent time point, precluding assessment of temporal dynamics in microbiome responses. Third, we used different paradigms for prenatal and postnatal stresses which can impact comparisons. The prenatal stress did not induce changes in corticosterone at day 35 and—the three 45‐min daily restraint sessions could be lengthened to continuous single or multiple 3‐h restraints during the same time window (for review see Weinstock 2017); these models could be explored in future studies to determine whether differing stress durations influence lasting basal corticosterone and gut microbiome outcomes. Fourth, we did not measure maternal behaviour in the LBN paradigm; though previous work has demonstrated it to induce erratic behaviour in mothers and impacts on offspring (Guo et al. 2015) we cannot be certain what this might have involved such as unpredictable care or increased licking/grooming that impacted the offspring. Fifth, fluctuations in hormone levels, within‐individual animals such as occurring across the oestrous cycle, could also influence microbiome composition, further contributing to variability in host–microbe interactions. Sixth, we did not use a positive control and while we did use a negative control for the 16S amplification and saw no bands, we did not sequence this negative control. Lastly, the strong separation observed between cohorts, despite shared housing conditions, highlights the sensitivity of the gut microbiome to subtle environmental and experimental variation. Although animals were maintained within the same facility under controlled conditions such as light and temperature, cohort‐specific factors such as timing of experiments, cage‐level effects, and microenvironment differences may contribute to compositional divergence. Previous studies have demonstrated that cage effects alone can significantly influence microbiome structure, even under otherwise standardised conditions (Singh et al. 2021). To mitigate these influences, analyses were performed within cohorts using matched controls, ensuring that detected effects reflect treatment‐related changes rather than baseline cohort differences. Importantly the replication of stress‐associated signals across independent cohorts (e.g., POS) suggests that these effects are biologically meaningful and robust to compositional modelling approaches, rather than artefacts of environmental variability.

## 5 Conclusion

In summary, this study demonstrates that ELS induces model‐specific and sex‐dependent alterations in gut microbiome composition, which are primarily reflected at the level of community structure rather than individual taxa. DEXA exposure produces the most consistent compositional signal, particularly in males, whereas PRS shows minimal detectable effects and POS exhibits a more heterogeneous response characterised by increased dispersion and limited taxonomic changes.

More broadly, these findings illustrate how combining compositional beta‐diversity analyses with machine learning approaches can reveal reproducible microbiome ‘states’ associated with ELS, even in the absence of large taxonomic shifts. Applying similar frameworks in larger and longitudinal cohorts will be essential to determine how these subtle microbial signatures contribute to long‐term physiological outcomes.

## Author Contributions

**Ayomide Adetunji:** investigation. **Elliot F. Jennings:** methodology, software, formal analysis, data curation, writing – review and editing, visualization. **Hamilton Imongan:** investigation. **Chiamaka Vera Oguanya:** investigation. **Michael Harte:** conceptualization, writing – review and editing. **Rebecca Woods:** conceptualization, investigation, supervision, writing – review and editing, writing – original draft, formal analysis. **Laura Smith:** investigation. **Khairiah Almushri:** investigation. **Oriana Gamrot:** conceptualization, investigation. **Emmanuella Omuluche:** investigation. **Liam Hanson:** investigation, methodology. **Kubili John:** investigation. **Chris Murgatroyd:** conceptualization, methodology, investigation, writing – original draft, supervision, formal analysis.

## Funding

R.W. is funded through a BBSRC grant BB/W017598/1 and E.F.J. is funded for a studentship through the Cancer Prevention Research Trust both awarded to C.M. This work was further supported by internal institutional funds from Manchester Metropolitan University, including funding from MSc student projects and an internally funded PhD studentship.

## Conflicts of Interest

The authors declare no conflicts of interest.

## Supporting information


**Figure S1:** Rarefaction sensitivity analysis. (A) Alpha diversity metrics (Observed Genera, Shannon, Simpson) recalculated after rarefaction to a common read depth across PRS, POS and DEXA cohorts. No strong cohort‐level alpha‐diversity differences were observed after rarefaction. (B) Ordination of rarefied genus‐level profiles following CLR transformation, showing broad cohort‐level compositional separation was preserved after subsampling. Together, these analyses indicate that the main findings were not driven by sequencing depth variation.
**Figure S2:** Serum corticosterone levels of Prenatal (A) and Postnatal (B) stress and control male and female animals. Mean + SEM (*n* = 12 for each group). Significant *p* values are shown.
**Figure S3:** (A) Per‐sample sequencing depth summary (reads per sample). (B) Distribution of sequencing depth across all samples. (C) Distribution of mean read quality (Qscore). (D) Fraction of reads within the expected full length 16S amplicon range (1300–1700 bp). (E.) Distribution of unclassified gene‐level fraction across samples. Overall, sequencing depth and quality metrics were consistent across samples, with the majority of reads falling within the expected amplicon length and high mean Q‐scores, indicating robust sequencing performance suitable for downstream compositional analysis.

## Data Availability

Complete analysis scripts, intermediate count tables, are provided in the supporting information at https://github.com/ElliotJenningsPhD/16S‐ELS‐Microbiome.
