# Supplementary material for: Effect of Prenatal and Postnatal Stress in Rats on the Gut Microbiome in Adolescence
Source: J Neurochem. 2026 Jul 27;170(7):e70530. doi: 10.1111/jnc.70530 (PMC13408194; doi:10.1111/jnc.70530)
Supplement: Supplementary file 1 — Figure S1: Rarefaction sensitivity analysis. (A) Alpha diversity metrics (Observed Genera, Shannon, Simpson) recalculated after rarefaction to a common read depth across PRS, POS and DEXA cohorts. No strong cohort‐level alpha‐diversity differences were observed after rarefaction. (B) Ordination of rarefied genus‐level profiles following CLR transformation, showing broad cohort‐level compositional separation was preserved after subsampling. Together, these analyses indicate that the main findings were not driven by sequencing depth variation. Figure S2: Serum corticosterone levels of Prenatal (A) and Postnatal (B) stress and control male and female animals. Mean + SEM (n = 12 for each group). Significant p values are shown. Figure S3: (A) Per‐sample sequencing depth summary (reads per sample). (B) Distribution of sequencing depth across all samples. (C) Distribution of mean read quality (Qscore). (D) Fraction of reads within the expected full length 16S amplicon range (1300–1700 bp). (E.) Distribution of unclassified gene‐level fraction across samples. Overall, sequencing depth and quality metrics were consistent across samples, with the majority of reads falling within the expected amplicon length and high mean Q‐scores, indicating robust sequencing performance suitable for downstream compositional analysis. [file JNC-170-e70530-s001.pdf]

SUPPLEMENTARY INFORMATION

TITLE: Effect of prenatal and postnatal stress in rats on the gut microbiome in adolescence

AUTHORS: Rebecca Woods[1,2*] •, Elliot F Jennings [1*] • Laura Smith[1], Khairiah Almushri[3], Liam Hanson[1], Oriana Gamrot[1], Ayomide Adetunji[1], Chiamaka Vera Oguanya[1], Hamilton Imongan[1], Kubili John[1], Emmanuella Omuluche[1], Michael Harte[3], Chris Murgatroyd[1*]

[1]Department of Life Sciences, Manchester Met University

[2]Division of Evolution, Infection and Genomics, School of Biological Sciences, Faculty of Biology Medicine and Health, University of Manchester

[3]Division of Pharmacy and Optometry, School of Health Sciences, Faculty of Biology Medicine and Health, University of Manchester

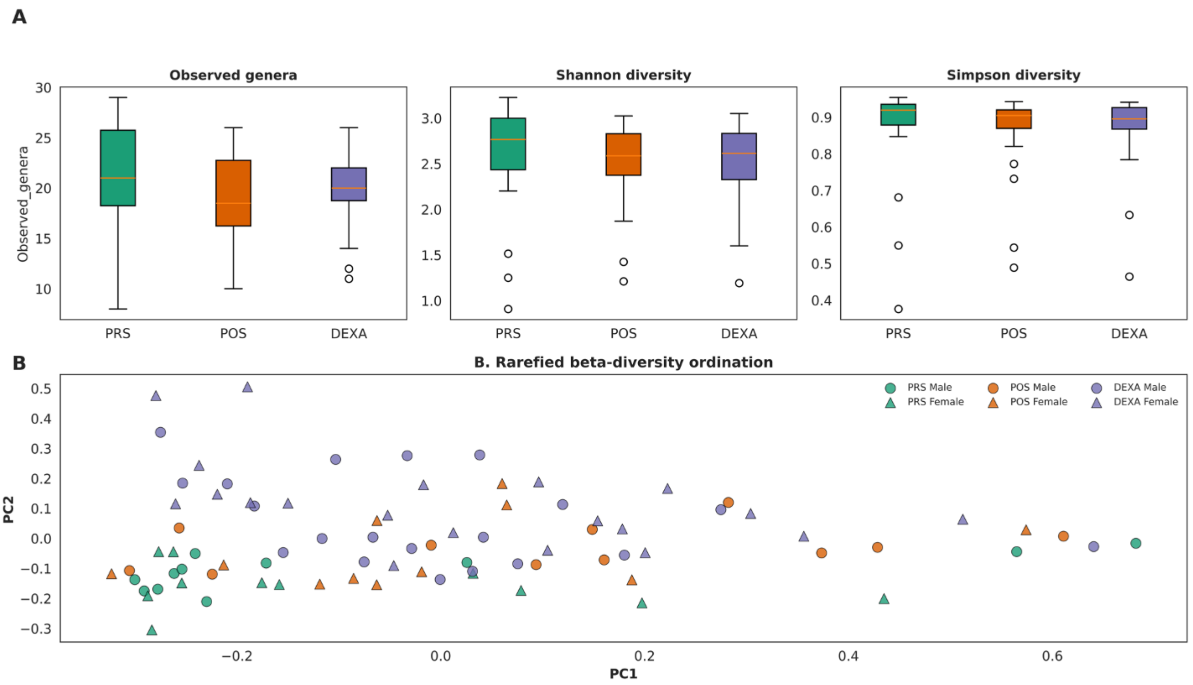

Figure S1: Rarefaction Sensitivity Analysis. (A) Alpha diversity metrics (Observed Genera, Shannon, Simpson) recalculated after rarefaction to a common read depth across PRS, POS, and DEXA cohorts. No strong cohort-level alpha-diversity differences were observed after rarefaction. (B) Ordination of rarefied genus-level profiles following CLR transformation, showing broad cohort-level compositional separation was preserved after subsampling. Together, these analyses indicate that the main findings were not driven by sequencing depth variation.

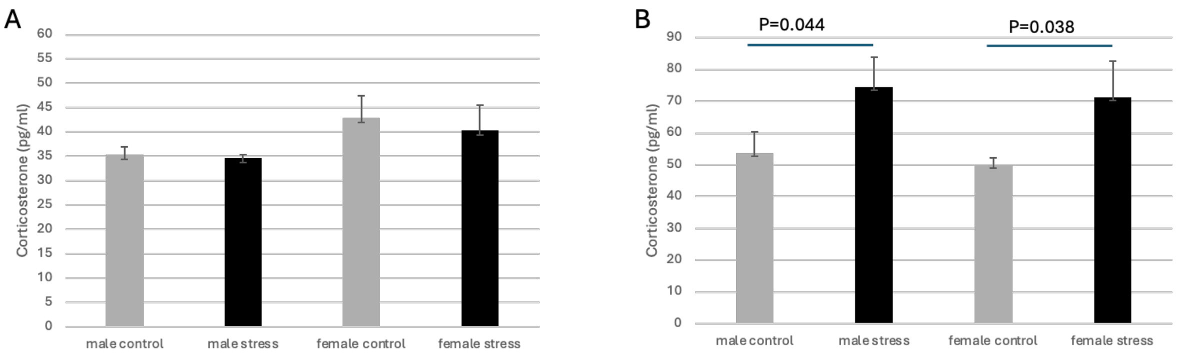

Figure S2: Serum corticosterone levels of Prenatal (A) and Postnatal (B) stress and control male and female animals. Mean + SEM (n = 12 for each group). Significant p values are shown.

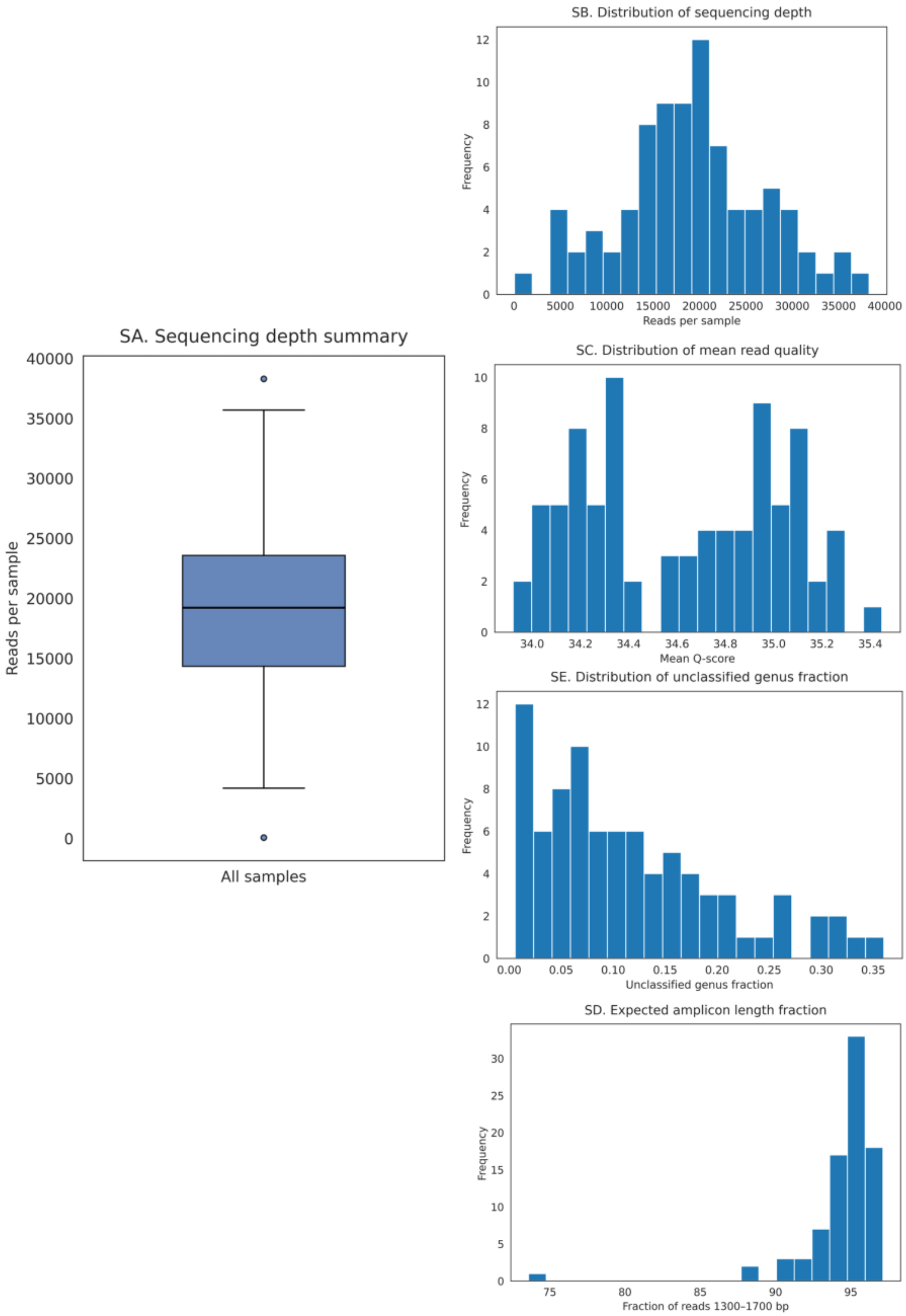

Figure S3: (A) Per-sample sequencing depth summary (reads per sample). (B) Distribution of sequencing depth across all samples. (C) Distribution of mean read quality (Q-score). (D) Fraction of reads within the expected full length 16S amplicon range (1,300-1,700 bp). (E.) Distribution of unclassified gene-level fraction across samples. Overall, sequencing depth and quality metrics were consistent across samples, with the majority of reads falling within the expected amplicon length and high mean Q-scores, indicating robust sequencing performance suitable for downstream compositional analysis
